# How Were Functional Somatic Symptoms in Children Influenced by the COVID-19 Pandemic? A Retrospective Study of Children Admitted to a Tertiary Pediatric Emergency Hospital in Bucharest, Romania

**DOI:** 10.3390/life16050713

**Published:** 2026-04-22

**Authors:** Daniela Păcurar, Alexandru Dinulescu, Andrei-Vlad Totu, Mirela-Luminița Pavelescu, Irina Dijmărescu

**Affiliations:** 1 Faculty of Medicine, Department of Pediatrics, “Carol Davila” University of Medicine and Pharmacy, 050474 Bucharest, Romania; daniela.pacurar@umfcd.ro (D.P.); andrei-vlad.totu0125@rez.umfcd.ro (A.-V.T.); irina.dijmarescu@umfcd.ro (I.D.); 2 Department of Pediatrics, Emergency Hospital for Children “Grigore Alexandrescu”, 011743 Bucharest, Romania

**Keywords:** functional somatic symptoms, somatization, psychosocial stressors, COVID-19 pandemic, children, adolescents

## Abstract

Background: Functional somatic symptoms (FSSs) represent a significant clinical challenge in pediatric populations, with prevalence estimates ranging from 10 to 30%. The COVID-19 pandemic introduced unprecedented psychosocial stressors that may have influenced the presentation and precipitating factors of these conditions. This article aimed to characterize the clinical presentations, precipitating factors, and temporal patterns of functional somatic disorders in children admitted to a tertiary pediatric hospital in Bucharest, Romania, across pre-pandemic (2017–2019), pandemic (2020–2022), and post-pandemic (2023–2025) periods. Methods: This retrospective study included 1043 patients aged 3–17 years admitted with somatic symptoms without identifiable organic pathology. Data were extracted using ICD-10 diagnostic codes and confirmed through individual chart review. Variables analyzed included demographics, symptoms, precipitating factors, symptom duration, and family psychiatric history. Results: Female patients predominated (67.0%), with a median age of 14 years. Cardiovascular symptoms were most frequent (43.9%), followed by neurological (30.4%), digestive (19.0%), and respiratory (6.3%) manifestations. Family-related factors (38.6%) and school-related stress (32.7%) were the primary precipitating factors. Significant pandemic-related differences emerged: medical-related precipitating factors increased during the pandemic (18.5% vs. 10.7% pre-pandemic, *p* < 0.001), respiratory symptoms were more frequent (9.8% vs. 5.9% pre-pandemic), and symptom duration before admission was significantly longer (median 1 vs. 1 pre-pandemic and 0 months post-pandemic; *p* < 0.001). Conclusions: The COVID-19 pandemic substantially influenced pediatric somatic presentations, with increased health anxiety, respiratory symptoms, and delayed healthcare-seeking behavior. Post-pandemic patterns suggest the persistent influence of traditional stressors alongside pandemic-related effects.

## 1. Introduction

Functional somatic symptoms (FSSs) refer to physical complaints that are not fully explained by identifiable organic pathology but are associated with significant distress or functional impairment [1,2,3]. In pediatric populations, these symptoms commonly include abdominal pain, headache, chest pain, or dyspnea and represent a frequent cause of healthcare utilization [4,5,6,7]. Although overlapping terminology exists in the literature, including somatoform disorders, somatic symptom disorder (SSD), and psychosomatic disorders, these concepts are not fully interchangeable. SSD, as defined in DSM-5, emphasizes excessive thoughts, feelings, or behaviors related to somatic symptoms, rather than the absence of a medical explanation [8,9]. In contrast, FSS is often used as a broader, clinically pragmatic term, particularly in pediatric settings where strict psychiatric classification may be less applicable [3,10]. It is estimated that the rate of FSSs in the general population is around 10%, and in children, the estimated rate is even higher (10–30%) [2,10].

FSSs share symptomatic overlap with psychiatric conditions and involve psychological stress worsening an existing physical condition, so they represent distinct clinical entities that require different approaches [11,12,13]. Psychiatric disorders such as autism spectrum disorder (ASD), attention-deficit/hyperactivity disorder (ADHD), anxiety disorders, and depression have been extensively characterized and are associated with well-defined behavioral and cognitive symptomatology, in contrast to psychosomatic disorders, which primarily manifest through somatic complaints, though psychological comorbidities frequently coexist [14,15,16,17,18,19].

The relationship between childhood trauma, particularly abuse and neglect, and FSSs is extensively documented in the literature [20,21,22,23,24]. Multiple somatic symptoms among children show higher odds of experiencing physical abuse, with abused children reporting significantly more functional or somatic disorders than controls and experiencing longer durations of functional problems [25,26,27,28]. Female pediatric patients more commonly internalize behavioral attitudes through depression, social withdrawal, and somatic symptoms, which are more commonly found in this gender group, while in male pediatric patients, externalization of emotions through aggression and conduct problems is more frequently described [29,30,31]. Childhood emotional abuse establishes patterns that persist into adulthood, with internalization of negative self-beliefs and maladaptive emotional regulation contributing to chronic somatic symptom experiences [32,33].

Aside from clinical and possible later developmental issues, the Coronavirus Disease 2019 (COVID-19) pandemic also influenced children’s mental health and somatic presentations globally, creating unprecedented stressors affecting family dynamics, educational continuity, social connections, and healthcare access [34,35,36,37,38,39,40,41]. Systematic reviews and meta-analyses have demonstrated a substantial increase in stress-related behaviors, anger, irritability, withdrawal symptoms, fear, anxiety, and elevated depressive symptoms, among children during pandemic periods [42,43,44,45,46]. A meta-analysis published by Racine et al. (2021) that included 80,879 pediatric subjects globally reported pooled prevalence estimates of clinically elevated depression and anxiety at 25.2% and 20.5%, respectively, approximately double the pre-pandemic rates [47]. Children quarantined during the pandemic demonstrated significantly higher rates of post-traumatic stress symptoms, anxiety, depression, and somatic complaints compared to non-quarantined ones [36]. The pandemic period also disrupted healthcare access, with documented decreases in diagnostic rates and reduced medical services [48,49,50].

While previous studies have documented the prevalence of functional somatic symptoms and their association with psychological distress, less is known about how precipitating factors and clinical presentations evolve over time in response to large-scale societal stressors such as the COVID-19 pandemic. In particular, longitudinal data comparing pre-pandemic, pandemic, and post-pandemic periods in pediatric emergency settings remain limited. Therefore, this study aims to characterize the clinical presentation, precipitating factors, and temporal trends of functional somatic symptoms in children admitted to a major tertiary pediatric hospital in Bucharest, Romania, with a specific focus on changes associated with the COVID-19 pandemic.

## 2. Materials and Methods

### 2.1. Study Design and Setting

This is a retrospective 9-year study that includes patients under 18 years old (3–17 years old) with FSSs admitted to the Pediatrics department of the tertiary Hospital for Children, Bucharest, Romania, from 1 February 2017 to 31 December 2025. The hospital is one of the largest pediatric hospitals in Romania, but it lacks a psychiatric department, as it is a hospital that mainly aims to treat organic diseases, especially emergencies. All patients were initially evaluated in an emergency setting and subsequently admitted to pediatric subspecialty departments (pediatrics, neurology, or cardiology) for further investigation and monitoring. All patients included in the study were investigated, and organic diseases were excluded, before being sent for a psychological/psychiatric examination.

### 2.2. Selection Criteria

The study group included patients with no organic disease after proper clinical and paraclinical evaluation, for whom a psychological/psychiatric examination identified elements that supported a somatic disorder. Patients with known chronic diseases (organic or psychiatric) or those with attempted suicide through intoxication with different substances were excluded.

### 2.3. Data Collection

**Figure 1:**
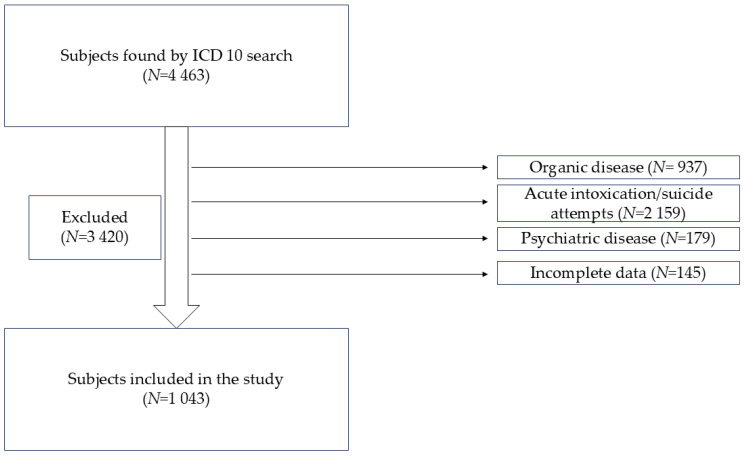
Data selection.

The patients were selected from the electronic register of the hospital based on International Classification of Diseases, 10th Revision (ICD-10) diagnosis codes. The following codes were used: F40.0 (Agoraphobia); F40.1 (Social phobias); F40.2 (Specific (isolated) phobias); F40.8 (Other phobic anxiety disorders); F40.9 (Phobic anxiety disorder, unspecified); F41.0 (Panic disorder [episodic paroxysmal anxiety]); F41.1 (Generalized anxiety disorder); F41.2 Mixed anxiety and depressive disorder; F41.3 (Other mixed anxiety disorders); F41.8 (Other specified anxiety disorders); F41.9 (Anxiety disorder, unspecified); F45.0 (Somatization disorder); F45.1 (Undifferentiated somatoform disorder); F45.2 (Hypochondriacal disorder); F45.3 (Somatoform autonomic dysfunction); F45.4 (Persistent somatoform pain disorder); F45.8 (Other somatoform disorders); F45.9 (Somatoform disorder, unspecified); F60.6 (Anxious [avoidant] personality disorder); F68.0 (Elaboration of physical symptoms for psychological reasons); F68.1 (Intentional production or feigning of symptoms or disabilities, either physical or psychological [factitious disorder]); F92.8 (Other mixed disorders of conduct and emotions); F93.0 (Separation anxiety disorder of childhood); F93.1 (Phobic anxiety disorder of childhood); F93.2 (Social anxiety disorder of childhood); F93.3 (Sibling rivalry disorder); F93.8 (Other childhood emotional disorders); F93.9 (Childhood emotional disorder, unspecified); R45 (Symptoms and signs involving emotional state); Z62 (Other problems related to upbringing). ICD-10 diagnostic codes were used solely as a screening tool to identify potentially eligible patients. Final inclusion was based on individual chart review, confirming the absence of organic pathology and the presence of clinical features consistent with somatic disorders (Figure 1).

After identifying the subjects who met the selection criteria, we collected the following variables: age, age group (patients were stratified into three age groups according to developmental stage: preschool-aged children (3–5 years), school-aged children (6–11 years), and adolescents (12–18 years)), sex, area of provenance, the dominant symptom and its category, duration from the onset to admission (months), number of admissions for the same symptoms, precipitating factors and a family history of psychiatric or psychological disorders. No standardized or validated questionnaire was used for data extraction. All variables were obtained retrospectively from patients’ medical records, following predefined criteria established by the research team to ensure consistency. Precipitating factors and symptom categories were identified based on clinical documentation and later grouped into broader categories for analysis.

The dominant symptom was defined as the main clinical manifestation that prompted hospital admission, as documented in the medical records. Symptoms were subsequently grouped into clinically relevant categories (cardiovascular, neurological, respiratory, or digestive), based on the prevailing presentation. Syncope and presyncope were classified as functional cardiovascular manifestations in the absence of structural cardiac or neurological pathology, even when a neurological assessment was performed during hospitalization. To contextualize the burden of somatic symptoms, the annual proportion of such admissions was calculated by dividing the number of eligible cases by the total number of pediatric hospital admissions for each study year (admissions for acute intoxications/poisoning/suicide attempts in the toxicology department were excluded from the denominator, as they represent a distinct clinical entity not comparable to functional or psychosomatic presentations).

We divided the study group into three periods, in relation to the COVID-19 pandemic: pre-pandemic (2017–2019), pandemic period (2020–2022), and post-pandemic (2023–2025). The three groups were compared.

Precipitating factors were categorized as school-related, family-related, social, or medical. Bullying was classified as a school-related precipitating factor. Family conflict was defined as recurrent disputes within the household. Parental divorce was defined as a legal or formal separation of parents in which at least one parent remained actively involved in the child’s daily care. Parental absence was defined as the absence of one or both biological parents due to death, migration, or long-term separation, resulting in the child being raised by extended family members or caregivers and was analyzed separately from parental divorce. Sibling rivalry was defined as emotional distress related to the birth or presence of a sibling, manifested by feelings of neglect, jealousy, or competition for parental attention. Health anxiety was defined as excessive worry about having or developing a serious illness, despite medical reassurance and the absence of organic disease. The death of a close person was defined as the death of a family member or a close friend, perceived by the patient as a significant emotional stressor. Illness of a close person was defined as the occurrence of an acute or chronic medical condition in a family member or close friend, perceived by the patient as a significant emotional stressor. Romantic relationship stress was defined as emotional distress related to difficulties, rejection, or a breakup within a close romantic relationship. Fear of public speaking was defined as fear or distress related to speaking in front of an audience that caused the symptomatology. An unidentified precipitating factor was recorded when no clear psychological, familial, school-related, or medical trigger could be identified from the available medical records. A family history of psychiatric or psychological disorders was defined as a documented history of mental health conditions in first-degree relatives.

The data was collected in Microsoft Office Excel 2024.

### 2.4. Statistical Analysis

The data was analyzed using IBM SPSS Statistics version 25 and illustrated using Microsoft Office Excel/Word 2024. Quantitative variables were tested for normal distribution using the Shapiro–Wilk test and are presented as medians with interquartile ranges (IQRs). Quantitative variables were tested between independent groups using Mann–Whitney U tests. The Kruskal–Wallis test was used to determine significant differences between two or more groups of an independent variable. Post hoc pairwise comparisons following the Kruskal–Wallis test were performed using Dunn’s test with Bonferroni-adjusted *p* values. Associations between categorical variables were analyzed using the Pearson chi-square test. For 2 × 2 tables or small sample sizes, or when expected cell counts were below 5, Fisher’s exact test (2-sided) was applied when computationally feasible. The threshold for statistical significance was considered a *p*-value under 0.05.

## 3. Results

### 3.1. Subject Characteristics

We found 1043 patients who met the selection criteria. Admissions were distributed across the entire study period, with the highest number of cases recorded in 2019, 177 (17.0%). When grouped according to the COVID-19 pandemic, 42.2% of cases occurred in the pre-pandemic period, 27.4% during the pandemic, and 30.4% in the post-pandemic period. Female patients predominated, accounting for 67.0% of the study population. The median age at admission was 14 years (11–16). Adolescents represented the majority of cases (73.3%), followed by school-aged children (25.0%), while preschool children were the least represented (1.7%). Regarding the area of provenance, urban residence predominated, with 699 patients (67.0%) originating from urban areas, compared to 344 (33.0%) from rural settings (Table 1).

### 3.2. Annual Statistics

The annual proportion of hospital admissions attributed to functional and somatic symptoms varied across the study period, ranging from 1.63% to 2.90%. The highest proportion was recorded in 2019 (2.90%), while lower proportions were observed in the later study years. During the pandemic period (2020–2021), somatic admissions accounted for 2.46% and 2.61% of all pediatric hospitalizations, respectively. In the post-pandemic period (2023–2025), the proportion remained below 2.0% each year (Table 2).

### 3.3. Symptomatology

Cardiovascular symptoms were the most frequently reported group in the study, accounting for 43.9% (*n* = 458) of all cases. Within this category, precordial pain was the most commonly reported symptom (*n* = 223), followed by syncope (*n* = 125), palpitations (*n* = 71), and presyncope (*n* = 39). Neurological symptoms represented 30.4% (*n* = 317) of presentations. The most frequent neurological manifestation was headache (*n* = 227), while other reported symptoms included non-epileptic involuntary movements (n = 38), paresthesia (n = 30), gait disturbances (*n* = 11), vertigo (*n* = 10), and insomnia (*n* = 1). Digestive manifestations accounted for 19.0% (n = 198) of cases, with abdominal pain being the predominant symptom (*n* = 124). Less frequently reported digestive symptoms included inappetence (*n* = 30), vomiting (*n* = 24), nausea (*n* = 6), dysphagia (*n* = 5), constipation (*n* = 4), diarrhea (*n* = 3), and encopresis (*n* = 2). Respiratory symptoms were observed in 6.3% (*n* = 66) of patients, predominantly dyspnea (*n* = 62), while cough was rarely reported (*n* = 4). Urinary manifestations were uncommon, with enuresis recorded in four patients (0.4%). The summary of these symptoms can be found in Table 3.

Family-related precipitating factors were the most frequently identified, accounting for 38.6% (*n* = 403) of cases. Within this group, family conflict was the predominant factor (*n* = 352), while parental divorce (*n* = 24), parental absence (*n* = 18), and sibling rivalry (*n* = 9) were less commonly reported. School-related factors represented 32.7% (*n* = 341) of cases, with academic stress (*n* = 214) being the most frequently documented school-related precipitant, followed by bullying (*n* = 127). Medical-related factors were identified in 11.6% (*n* = 121) of cases. The most commonly reported medical-related factors were health anxiety (*n* = 49), the death of a close person (*n* = 40), and the illness of a close person (*n* = 32). Social-related precipitating factors were less frequent, accounting for 2.6% (*n* = 27) of cases. These included romantic relationship stress (*n* = 25) and fear of public speaking (*n* = 2). In 14.5% (*n* = 151) of patients, no identifiable precipitating factor was documented (Table 4).

### 3.4. Precipitating Factors

Sixty-six (6.3%) of patients had a family history of psychiatric or psychological disorders.

### 3.5. Pandemic-Related Differences

There was no statistical difference in the median age of patients among the three periods (*p* = 0.369). There was no difference by sex (*p* = 0.439) or area of provenance (*p* = 0.395).

The distribution of symptom groups differed significantly across the three study periods (*p* = 0.014) (Table 5). Cardiovascular symptoms represented the most frequent symptom group in all periods; however, their relative proportion varied over time. During the COVID-19 pandemic, cardiovascular symptoms accounted for a lower proportion of admissions (39.5%) compared with the pre-pandemic (45.5%) and post-pandemic periods (45.7%). In contrast, digestive symptoms showed a relative increase during the pandemic period (23.1%), compared with the pre-pandemic (16.8%) and post-pandemic periods (18.3%). Neurological symptoms remained relatively stable across all periods, accounting for approximately one-third of cases. Respiratory symptoms were more frequent during the pandemic period (9.8%) than in the pre-pandemic (5.9%) and post-pandemic periods (3.8%). Urinary symptoms were rare in all periods.

The distribution of precipitating factor types differed significantly across the three study periods (*p* < 0.001) (Table 6). While family-related and school-related factors remained the most frequent precipitating factors throughout all periods, notable shifts were observed in the contribution of medical-related factors. Especially during the COVID-19 pandemic, the proportion of cases with medical-related precipitating factors increased markedly to 18.5%, compared with 10.7% in the pre-pandemic period and 6.6% in the post-pandemic period. This represented the highest relative contribution of medical-related factors across the entire study interval. In contrast, family-related factors showed relatively stable proportions during the pre-pandemic (41.4%) and pandemic periods (40.6%), followed by a moderate decrease in the post-pandemic period (33.1%). School-related factors demonstrated comparable proportions during the pandemic (34.6%) and post-pandemic periods (33.8%).

The duration of symptoms differed significantly across the three study periods (*p* < 0.001). The longest symptom duration was observed among patients admitted during the COVID-19 pandemic (1 month (0–4) vs. 1 month (0–3.5) pre-pandemic and 0 months (0–1) post-pandemic).

The specific familial factor was compared across the three periods. Across all periods, family conflict was the most frequently reported trigger, accounting for 87.3% of all cases (352/403). Divorce-related conflicts were more frequently observed during the COVID-19 period (11.2%) compared with the pre-COVID-19 (5.5%) and post-COVID-19 periods (1.0%) (*p* < 0.001) (Table 7).

Comparing the medical factor across time, health anxiety was the predominant factor across all three periods. Even though health anxiety had a higher rate during the COVID-19 period (47.2% vs. 34% pre-pandemic and 38.1% post-pandemic), the difference was not statistically significant (*p* = 0.355) (Table 8).

Comparing school-related factors, across all three periods, academic stress remained the predominant trigger: 58.5% in the pre-COVID-19 period, 68.7% during the COVID-19 period, and 62.6% in the post-COVID-19 period. Bullying showed a relatively stable distribution, representing 41.5% in the pre-COVID-19 period, 31.3% during COVID, and 37.4% in the post-COVID-19 period. There were no statistically significant differences across periods (*p* = 0.284) (Table 9).

## 4. Discussion

Our study documents that FSSs accounted for 1.63% to 2.90% of total pediatric hospital admissions over the nine-year study period (2017–2025), with considerable year-to-year variation. Community-based studies typically report higher prevalence rates: functional somatic symptoms occur in 4.4% of people aged 15–24 years in general practice settings, with the actual rate in the general pediatric population being reported as higher, 10–30% [51,52,53]. The difference between the reported community prevalence and our observed hospital admission rate may suggest that most children with functional symptoms are managed in outpatient settings, with only those experiencing severe, persistent, or alarming symptoms needing hospitalization. Our tertiary emergency hospital setting naturally selects for more acute presentations, which may explain the relatively lower proportion compared to population-based estimates. In our cohort, digestive symptoms were frequently reported, particularly abdominal pain. Such presentations require careful differentiation between functional disorders and common organic conditions, including gastrointestinal infections, constipation, food-related disorders, and Helicobacter pylori infection, reinforcing the importance of thorough clinical evaluation in pediatric emergency settings [54,55,56,57]. The predominance of cardiovascular and neurological symptoms in our cohort may reflect underlying mechanisms of autonomic dysregulation and altered interoceptive processing in children exposed to chronic stress. Cardiovascular complaints such as chest pain, palpitations, or syncope are frequently described in association with heightened sympathetic activation and increased autonomic lability, particularly in the context of anxiety and emotional distress [58,59]. Neurological symptoms, including headache, dizziness, or functional motor manifestations, may represent parallel expressions of central nervous system sensitivity to stress, involving altered pain perception, cortical arousal, and impaired emotional regulation [60,61,62].

The COVID-19 pandemic’s impact on FSS presentations manifested through several distinct patterns in our study. The heightened awareness of respiratory symptoms during and after the COVID-19 pandemic likely contributed to the relative increase in respiratory presentations during the pandemic period (9.8% versus 5.9% pre-pandemic) observed in our study. Health anxiety related to COVID-19, fear of infection, and increased attention to respiratory symptoms created a context where functional respiratory complaints may have been amplified or more readily brought to medical attention [63,64,65].

The marked increase in medical-related precipitating factors during the pandemic period (18.5% versus 10.7% pre-pandemic and 6.6% post-pandemic) may reflect increased health and death anxiety, fear of infection, and stress related to illness in family members. Health anxiety, defined as excessive worry about having or developing serious illness despite medical reassurance, intensified during the pandemic as children and families confronted unprecedented mortality risks, hospitalization of family members, disrupted healthcare access, and pervasive media coverage of COVID-19 severity [66,67]. The longer symptom duration until hospital admission observed during the pandemic period in our study could suggest delayed healthcare-seeking behavior and restricted healthcare access. Systematic studies document decreased diagnostic rates, reduced medical service utilization, and delays in presentation for non-COVID-19 conditions during pandemic periods [68,69,70]. The disruption of routine pediatric care, including healthy children’s visits and school health screenings that might have identified concerning symptoms earlier, might have contributed to delayed recognition. Healthcare systems themselves experienced significant strain during pandemic surges, with prolonged waiting times for non-urgent evaluations, reduced outpatient clinic capacity due to infection control measures, and reallocation of pediatric resources to the COVID-19 response. The expansion of telemedicine during the pandemic, while providing some access, may have been less effective for evaluating somatic complaints requiring physical examination, potentially delaying appropriate assessment and referral [71,72]. The heightened health anxiety observed during the COVID-19 pandemic has also been associated with a paradoxical increase in vaccine hesitancy. While fear of infection initially promoted interest in preventive measures, prolonged exposure to uncertainty, misinformation, and conflicting public health messages could have contributed to distrust in medical interventions. This phenomenon extended beyond COVID-19 vaccines to include routine childhood immunizations, with declining confidence reported for vaccines against measles and pertussis. As a result, health anxiety during the pandemic did not uniformly translate into protective health behaviors but, in some cases, fostered avoidance and skepticism, ultimately posing a risk for the resurgence of vaccine-preventable diseases [73,74,75,76,77,78].

The pandemic’s psychological impact on children involved multiple stressors beyond health anxiety. School closures and transitions to remote learning disrupted educational routines, reduced social interactions, eliminated structured daily schedules, and created academic uncertainties [79]. The relative stability of family-related precipitating factors across all periods (41.4% pre-pandemic, 40.6% pandemic, 33.1% post-pandemic) suggests that fundamental family stressors remained prominent regardless of the pandemic context. However, the pandemic likely intensified pre-existing family vulnerabilities through multiple mechanisms. Parental stress related to job loss, financial instability, childcare responsibilities, and homeschooling demands could have been factors that increased tension in families [80,81]. Our findings showing a significantly higher rate of divorce-related conflicts during the COVID-19 period (11.2%) compared with pre-pandemic (5.5%) and post-pandemic (1%) periods, *p* < 0.001, are consistent with previous reports indicating that pandemic-related stressors, social isolation, and economic instability significantly affected marital relationships and increased the risk of family conflict and divorce [82,83]. The persistently increased proportions of family-related and school-related precipitants in the post-pandemic period suggest that traditional stressors remained cardinal despite pandemic influence.

The post-pandemic period (2023–2025) in our study reveals interesting patterns suggesting both normalization and persistent effects. The decrease in medical-related precipitating factors to 6.6% indicates a reduction in acute health anxiety. However, the sustained elevation of family-related factors (33.1%) and school-related factors (33.8%) suggests that traditional stressors have reasserted prominence, possibly enhanced by pandemic-period disruptions. The economic aftermath of the pandemic, including inflation, persistent financial insecurity, and continued employment instability, may have contributed to ongoing family stress [84,85].

The educational system continues to address learning losses and social–emotional impacts from pandemic disruptions, potentially maintaining school as a significant source of stress for many children [86,87].

The observed patterns may be understood within a broader framework of emotional dysregulation and somatic expression of psychological distress. In children and adolescents, a limited capacity for emotional awareness and regulation can often lead to the externalization of distress through bodily symptoms [88,89,90]. Pandemic-related stressors, including uncertainty, fear of illness, and the disruption of daily routines, may have amplified internalizing processes such as anxiety, which in turn manifested as somatic complaints [91]. This perspective fits with recent psychosomatic models showing that stress can influence how children experience and interpret bodily sensations, often making physical symptoms feel more intense or more concerning [92,93].

Unlike many previous studies focusing on self-reported somatic symptoms, our study evaluated somatic disorders with an impact on daily functioning and healthcare addressability, including hospitalization. The higher severity observed in patients with longer symptom duration before admission underscores the disruptive effect of the pandemic on children’s mental and physical well-being.

### Study Limitations

The use of ICD-10 codes as initial screening may have missed cases coded under alternative diagnoses, as well as the inclusion of a heterogeneous group of patients within the same diagnostic categories. This is especially relevant in children, where anxiety disorders and functional somatic symptoms often overlap. As a result, patients with potentially different underlying psychological mechanisms may have been grouped together, reflecting the limitations of relying on administrative coding in this context.

The single-center design in an urban tertiary emergency hospital limits generalizability to other settings, such as primary care, rural contexts, or outpatient specialty clinics, where presentation patterns, patient demographics, and available resources differ substantially. Given the emergency hospital setting, only patients requiring inpatient admission for urgent evaluation or monitoring were included. Consequently, milder functional somatic symptoms typically managed in outpatient settings are likely underrepresented. The inclusion of preschool children poses a conceptual challenge, as formal diagnostic criteria for somatic symptom disorder are not well established in this age group. Nevertheless, these cases were retained to reflect early, developmentally mediated functional somatic presentations frequently encountered in pediatric emergency settings.

## 5. Conclusions

In the COVID-19 pandemic period, among children with FSSs, respiratory complaints were more frequent, symptom duration until hospital admission was longer, and medical anxiety was more frequent as a precipitating factor, while traditional family and school stressors remained persistently influential. The divorce-related stress rate was higher in the pandemic period. The lessons learned from pandemic-period changes in FSS presentations should inform healthcare system planning for future public health crises and their associated mental health consequences for children and adolescents.

## Figures and Tables

**Table 1:** Descriptive statistics of the population’s main characteristics.

Variable	*N* (%)	Median (IQR)
**Year of admission**		
2017	109 (10.5%)	
2018	154 (14.8%)	
2019	177 (17%)	
2020	83 (8%)	
2021	102 (9.8%)	
2022	101 (9.7%)	
2023	123 (11.8%)	
2024	96 (9.2%)	
2025	98 (9.4%)	
**Period in relation to the COVID-19 pandemic**		
Pre-pandemic (2017–2019)	440 (42.2%)	
During pandemic (2020–2022)	286 (27.4%)	
Post-pandemic (2023–2025)	317 (30.4%)	
**Sex**		
Male	344 (33%)	
Female	699 (67%)	
**Age (years)**		
		14 (11–16)
Preschool children (2–5 years)	18 (1.7%)	
School-aged children (6–11 years)	261 (25%)	
Adolescents (12–18 years)	764 (73.3%)	
**Area of provenance**		
Urban	699 (67%)	
Rural	344 (33%)	

**Table 2:** Annual proportion of somatic admissions.

Year	Somatic Admissions (*N*)	Total Pediatric Admissions (*N*)	Proportion (%)
2017	109	5236	2.08%
2018	154	5772	2.67%
2019	177	6113	2.90%
2020	83	3377	2.46%
2021	102	3904	2.61%
2022	101	6214	1.63%
2023	123	6215	1.98%
2024	96	5786	1.66%
2025	98	5882	1.67%

**Table 3:** Distribution of predominant symptoms and their clinical group.

Symptom Group	Symptom	*N*	Total
Cardiovascular	Presyncope	39	458 (43.9%)
	Syncope	125	
	Precordialgia	223	
	Palpitations	71	
Neurological	Headache	227	317 (30.4%)
	Non-epileptic involuntary movements	38	
	Insomnia	1	
	Gait disturbances	11	
	Vertigo	10	
	Paresthesia	30	
Digestive	Abdominal pain	124	198 (19%)
	Constipation	4	
	Diarrhea	3	
	Dysphagia	5	
	Encopresis	2	
	Inappetence	30	
	Nausea	6	
	Vomiting	24	
Respiratory	Cough	4	66 (6.3%)
	Dyspnea	62	
Urinary	Enuresis	4	4 (0.4%)

**Table 4:** Distribution of precipitating factors and their group.

Precipitating Factor Group	Precipitating Factor	*N*	Total
Family-related	Family conflict	352	403 (38.6%)
	Divorce	24	
	Parental absence	18	
	Sibling rivalry	9	
School-related	Academic stress	214	341 (32.7%)
	Bullying	127	
Medical	Health anxiety	49	121 (11.6%)
	Death of a close person	40	
	Illness of a close person	32	
Social	Romantic relationship stress	25	27 (2.6%)
	Fear of public speaking	2	
Unidentified precipitating factor	Not found	151	151 (14.5%)

**Table 5:** Distribution of symptom groups by COVID-19 period.

Period	Cardiovascular *n* (%)	Digestive *n* (%)	Neurological *n* (%)	Respiratory *n* (%)	Urinary *n* (%)	Pearson Chi-Square Test (2-Sided) (*p*)
Pre-pandemic (2017–2019)	200 (45.5%)	74 (16.8%)	139 (31.6%)	26 (5.9%)	1 (0.2%)	=0.014
Pandemic (2020–2022)	113 (39.5%)	66 (23.1%)	79 (27.6%)	28 (9.8%)	0	
Post-pandemic (2023–2025)	145 (45.7%)	58 (18.3%)	99 (31.2%)	12 (3.8%)	3 (0.9%)	

**Table 6:** Distribution of precipitating factor types by COVID-19 period.

Period	Familial *n* (%)	Medical *n* (%)	School-Related *n* (%)	Social *n* (%)	Unidentified *n* (%)	Pearson Chi-Square Test (2-Sided) (*p*)
Pre-pandemic (2017–2019)	182 (41.4%)	47 (10.7%)	135 (30.7%)	8 (1.8%)	68 (15.5%)	<0.001
Pandemic (2020–2022)	116 (40.6%)	53 (18.5%)	99 (34.6%)	1 (0.3%)	17 (5.9%)	
Post-pandemic (2023–2025)	105 (33.1%)	21 (6.6%)	107 (33.8%)	18 (5.7%)	66 (20.8%)	

**Table 7:** Distribution of familial factor types by COVID-19 period.

Period	Family Conflict *n* (%)	Divorce *n* (%)	Family Abandonment *n* (%)	Sibling Rivalry *n* (%)	Fisher’s Exact Test (2-Sided) (*p*)
Pre-pandemic (2017–2019)	156 (85.7%)	10 (5.5%)	11 (6.0%)	5 (2.7%)	<0.001
Pandemic (2020–2022)	93 (80.2%)	13 (11.2%)	7 (6.0%)	3 (2.6%)	
Post-pandemic (2023–2025)	103 (98.1%)	1 (1.0%)	0	1 (1.0%)	

**Table 8:** Distribution of medical factor types by COVID-19 period.

Period	Health Anxiety *n* (%)	Death of a Close Person *n* (%)	Illness of a Close Person *n* (%)	Fisher’s Exact Test (2-Sided) (*p*)
Pre-pandemic (2017–2019)	16 (34.0%)	20 (42.6%)	11 (23.4%)	=0.355
Pandemic (2020–2022)	25 (47.2%)	15 (28.3%)	13 (24.5%)	
Post-pandemic (2023–2025)	8 (38.1%)	5 (23.8%)	8 (38.1%)	

**Table 9:** Distribution of school-related factor types by COVID-19 period.

Period	Academic Stress *n* (%)	Bullying *n* (%)	Fisher’s Exact Test (2-Sided) (*p*)
Pre-pandemic (2017–2019)	79 (58.5%)	56 (41.5%)	=0.284
Pandemic (2020–2022)	68 (68.7%)	31 (31.3%)	
Post-pandemic (2023–2025)	67 (62.6%)	40 (37.4%)	

## Data Availability

Data are contained within the article.

## Abbreviations

The following abbreviations are used in this manuscript:

FSS	Functional somatic symptoms
ASD	Autism spectrum disorder
COVID-19	Coronavirus disease 2019
ADHD	Attention-deficit/hyperactivity disorder
